# Stunting, selenium deficiency and anemia are associated with poor cognitive performance in preschool children from rural Ethiopia

**DOI:** 10.1186/s12937-016-0155-z

**Published:** 2016-04-12

**Authors:** Dawd Gashu, Barbara J. Stoecker, Karim Bougma, Abdulaziz Adish, Gulelat D. Haki, Grace S. Marquis

**Affiliations:** 1 Center for Food Science and Nutrition, Addis Ababa University, P. O. Box 1176, Addis Ababa, Ethiopia; 2 Department of Nutritional Sciences, Oklahoma State University, Stillwater, OK USA; 3 School of Dietetics and Human Nutrition, McGill University, 21111 Lakeshore Road, CINE Building, Sainte Anne-de-Bellevue, QC H9X 3 V9 Canada; 4 Micronutrient Initiative Africa, Addis Ababa, Ethiopia; 5 Department of Food Science and Technology, University of Botswana, Botswana College of Agriculture, Private Bag 0027, Gaborone, Botswana

**Keywords:** Anthropometry, Iron, Anemia, Selenium, Cognition, Children

## Abstract

**Background:**

Anthropometric characteristics and iron status affect cognitive performance in children. In addition, selenium can influence cognitive outcomes; protection of the brain from oxidative stress and its role in thyroid hormone metabolism are putative mechanisms.

**Methods:**

To investigate their association with cognitive performance, anthropometric indicators, iron biomarkers, and serum selenium of children (*n* = 541) of 54-60mo of age from rural Ethiopia were assessed. Cognitive assessment was conducted with the administration of two reasoning subtests of the Wechsler Preschool and Primary Scale of Intelligence and the school readiness test.

**Results:**

Stunting was found in 41.4 % of children, 28.7 % were underweight, and 6.3 % were wasted. The mean score of stunted children was lower than that of non-stunted children on non-verbal reasoning (7.0 ± 3.2vs7.9 ± 3.1; *p* = 0.01) and the school readiness tests (4.3 ± 2.2 vs 3.3 ± 2.1; *p* < 0.001). Compared to non-anemic children, anemic children had lower score for the verbal reasoning test (9.5 ± 1.7 vs 8.9 ± 2.2; *p* = 0.02). However, except for hemoglobin, none of the iron biomarkers had significant associations with the cognitive score of the study children (*p* > 0.05). Selenium deficient children had lower scores on all cognitive tests than normal children (*p* < 0.05).

**Conclusion:**

The present study finding linking chronic undernutrition and micronutrient deficiency to cognitive deficits suggests the need for designing effective intervention programmes to control for protein energy malnutrition and micronutrient deficiency and address cognitive development in children.

## Introduction

Protein energy malnutrition and micronutrient deficiencies are major concerns among children from low income countries. Worldwide, 25 % of children under 5 years are stunted, 15 % are underweight and 8 % are wasted [1]. Sub-Saharan Africa has the second largest number of stunted and underweight children next to south Asia [2]. In Ethiopia, 40.4 % of children under the age of five years are stunted, 25 % are underweight, and 9 % are wasted [3]. Iron deficiency is one of the world’s common single nutrient deficiencies [4] and about a quarter of the world’s populations are affected by anemia [5]. Selenium (Se) deficiency is less commonly studied and reported than anemia. Yet more than half a billion people in the world [6] and about 60 % of children from the northwest of Ethiopia [7] are reported to be Se deficient.

Protein-energy malnutrition during early childhood constrains structural and functional development of the brain and affects cognitive function [8]. Effects of early stunting on cognitive deficits can persist throughout later life [9] and successive generations [10]. Iron plays an important role for the synthesis of neurotransmitters and myelination of neurons [11]. Deficiency of iron has been associated with poorer cognitive performance in children and was implicated in long lasting effects even after treatment for iron deficiency [12, 13]. Anemia is also a significant risk factor for cognitive deficit [14].

Brain oxidative stress may be another significant contributor to cognitive deficits [15]. Selenium, a potent antioxidant micronutrient [16], may impact cognitive function by protecting the brain from oxidative damage [17]. In addition, Se is known to influence the process of synaptogenesis, myelination, and neuronal cell differentiation by regulating thyroid hormones [18]. Several researchers have investigated the association between Se nutrition and cognitive performance or protective effects of Se against cognitive deficit [19–23]. However, studies regarding the association of Se status and cognitive performance to date have been limited to animal models or related to age-induced memory loss in humans. This study investigated the association of anthropometric characteristics, iron biomarkers, and serum Se with cognitive performance of preschool children from rural Ethiopia.

## Materials and methods

### Study subjects

As part of a randomized control trial(ClinicalTrials.gov: NCT013496) investigating the effect of iodized salt on cognitive development of children, sixty rural kebeles from randomly selected six zones (West Gojjam, East Gojjam, North Gondar, North Wollo, South Wollo, and Wagehmera) of the Amhara region first were randomly selected. Next, one village was randomly selected from each of the sixty rural kebeles. A house-to-house census was conducted to register all children 6–60 mo old. Each child’s age was obtained from the immunization card or from the parents or guardian. An event calendar, containing dates and year of local or national festivals, traditions, and other major events, was prepared as an aide to help parents correctly report the age of their child. To avoid variation of cognitive performance due to difference in the age of children, the children were further classified into three groups as 6–10, 18–22 and 54-60mo. In the present analysis, baseline data were included from children (*N* = 541), 54-60mo of age from 26 villages randomly selected out of the original 60 villages.

### Anthropometric measurement

Height was measured in an erect position using a calibrated wooden height-measuring board (Stadiometer, Shorr Productions, Olney, MD, USA) with a sliding head bar while children were barefoot. Their height was measured in duplicate and recorded to the nearest 0.1 cm or in triplicate whenever the deviation between the first two measurements was > 0.5 cm and the average of all measurements was taken. Weight was measured using a battery-powered digital scale (Mother-child scale, Max capacity 440 lb (200 kg), precision 0.1 kg (TANITA WB-100, Tanita Corporation, Arlington Heights, IL,USA) while children wore light clothes with empty pockets and no shoes. The weight scale was calibrated at least twice a day. Weight was measured twice and recorded to the nearest 0.1 kg and the average was taken.

### Blood collection and analysis

Experienced nurses drew blood. Blood was drawn from the antecubital vein with disposable butterfly vacutainer needles (BD Vacutainer butterfly needles, 23G, 3/4", Franklin Lakes, NJ, USA) using standard safety measures. Hemoglobin (Hb) level was measured using a digital Hemocue (201). Serum ferritin was analyzed using a clinical analyzer, Electrochemilumenescense Immuno Assay (ECLIA) Elecsys2010 analyzer, Cobas e 411. Soluble transferrin receptor and α_1_-acid glycoprotein (AGP) was determined by a clinical chemistry analyzer Cobas Integra 400 system. The detailed procedure followed for the analysis of Hb, serum ferritin, serum soluble transferrin receptor (sTFR), and AGP is reported elsewhere [24]. Duplicate frozen serum samples were shipped to the laboratory of the Department of Nutritional Sciences at Oklahoma State University, USA in an insulated box with dry ice for Se analysis.

Serum Se was analyzed by inductively coupled plasma mass spectrometer (ICP-MS) (PerkinElmer, ELAN9000, Norwalk, CT). Serum samples were diluted in 0.1 % trace metal grade HNO_3_ (Fisher Scientific, Fair Lawn, NJ). Working standards for Se were freshly prepared by diluting 1000 ppm pure Se stock solution (PerkinElmer) in a solution of 0.1 % HNO_3_ and 0.5 % Triton-X-100 (Sigma Aldrich, St. Louis, MO). Gallium (PerkinElmer) was used as an internal standard. A reference standard of freeze-dried human serum (Utak Laboratories, Inc., Valencia, CA) was used to verify the method performance. The reference standard was analyzed within every twenty samples to check the performance of the instrument. The Se value (105.13-112.66 μg/l) of the reference serum from the ICP-MS analysis was near the target value (111 μg/l) set by the manufacturer. The inter- and intra- assay coefficient of variation for the analysis was 4.3 % and 1.2 %, respectively. Trace mineral free gloves, plastic ware and pipette tips were used during analysis of the serum samples. A serum Se concentration below 70.0 μg/l was defined as Se deficiency [25].

### Assessment of cognitive function

The cognitive assessment included two subtests of the Wechsler Preschool and Primary Scale of Intelligence (WPPSI-III) [26] and a 25-item school readiness test. The first two are considered sufficient for research purposes to measure fluid intelligence: Matrix Reasoning measures nonverbal analytic reasoning based on the ability to complete patterns and analogies; Similarities measures verbal reasoning based on the ability to form verbal concepts. The school readiness test assesses the acquisition of preliteracy and prenumeracy skills in preparation for primary school. These tests assign 1 point for every correct answer and 0 for incorrect answers. The cognitive tests were translated to the local language (Amharic) and back-translated to English to check for accuracy of translation. The tests were adapted to the context of the study children by replacing items and expressions in the original cognitive tests that are not familiar in the study area with elements common in the locality but with similar level of complexity and meaning. The cognitive tests were pretested in non-study children from neighboring rural kebeles and necessary corrections were made. The tests were conducted in Amharic. Psychology graduates were trained by experienced researchers to administer the tests in a child-friendly manner. Inter-rater reliability yielded highly significant correlations (*p* < 0.001). Before starting the test, research assistants made sure that children were at ease and relaxed so as to avoid fear and shyness. The tests were conducted mainly in the child’s home. We present here baseline standardized WPPSI scores and raw scores of the school readiness test.

### Statistical analysis

Statistical analysis of data was performed using SPSS for Windows (v18). Normality of data was checked by Kolmogorov-Smirnov test. WHO Anthro software was used for analysis of anthropometric measures. Height-for-age, weight-for-age, and weight-for-height were used to classify the study children into categories of nutritional status using the WHO Multicenter Growth Reference [27]. Children below −2 height-for-age z-score (HAZ), −2 weight-for-age z-score (WAZ), and −2 weight-for-height z-score (WHZ) were classified as stunted, underweight, and wasted, respectively. Pearson correlation was conducted to study bivariate correlations. Scores for the three cognitive tests of Se deficient and normal children, stunted and non-stunted, underweight and normal, and anemic and non-anemic children were compared using Student’s t-test. To see the effect of multiple nutrient deficiencies on cognitive performance compared to single nutrient deficiencies, children were classified into groups and their cognitive scores were compared using one way analysis of variance. The least significant difference method was used for mean separation. A probability level of *p* < 0.05 was considered statistically significant. Multiple regression was conducted to identify the most important variables (among micronutrients and anthropometric characteristics) influencing cognitive performance of the children. In addition, linear regression analysis was also done to study the association of several factors including mother’s education, family assets, child’s sex, dietary diversity, presence of goiter, urinary iodine, serum thyroxin, serum triodothyronine, serum thyroglobulin, and serum zinc with cognitive scores by children. However, they were either not significant or explained very small percentage of variability.

### Ethics

This study was conducted according to the guidelines laid down in the Declaration of Helsinki and all procedures involving human subjects were approved by the National Health Research Ethics Review Committee at the Ethiopian Science and Technology Commission, and the Institutional Review Boards at McGill University, Canada and Oklahoma State University, USA. Written informed consent was obtained from all parents or guardians of the study children.

## Results

The study children (*n* = 541) had male to female ratio of 1.02:1. The mean age of the children was 56.9 ± 1.8 mo with a range of 54 to 60 mo. Summary of HAZ, WAZ, and WHZ of study children is shown in Table 1. Bilateral edema was not evident in any of the children. Overall, 41.4 % (*n* = 224) of children were stunted, 28.7 % (*n* = 155) were underweight, and 6.3 % (*n* = 34) were wasted (Table 1).

**Table 1:** Nutritional status of children (*n* = 541) from the Amhara region, Ethiopia, October 2011-May 2012

Variable	Minimum	Maximum	Mean	SD	Proportion of children < −2 Z-scores, %(n)	Proportion of children ≤ −3 Z-scores, %(n)
HAZ	−4.31	0.67	−1.54	0.85	41.4(224)	14.8(80)
WAZ	−5.24	1.48	−1.74	1.16	28.7(155)	5.4(29)
WHZ	−2.93	2.19	−0.72	0.84	6.3(34)	0.0(0)

HAZ, Height for Age Z-score, WAZ, Weight for Age Z-score; WHZ, Weight for Height Z-score

Our previous analyses indicated that anemia was present in 13.6 % of children. Based on serum ferritin, 9.1 % of children were iron deficient. Iron deficiency anemia (IDA) was seen in 5.3 % of children and 14.2 % of children had elevated sTFR (>5.0 mg/l) [24]. Of the total sample, 58.4 % (*n* = 316) of children had serum Se concentrations below70 μg/l.

**Fig. 1:**
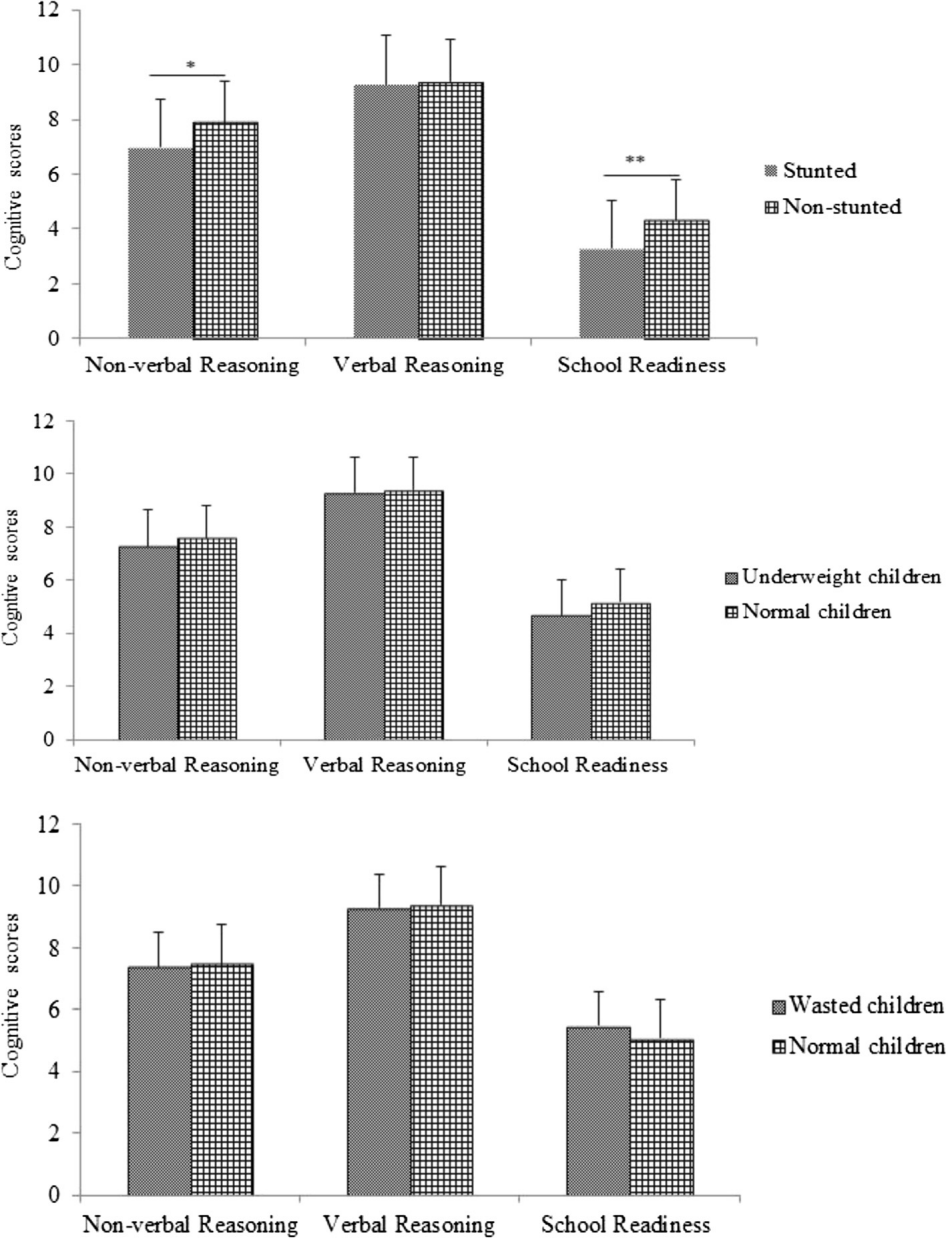
Comparison of cognitive scores of preschool children by nutritional status. * the difference is significant at *p* < 0.05, **the difference is significant at *p* < 0.01

A summary of cognitive scores of the study children is depicted in Table 2. The mean score of stunted children was significantly lower than mean score of non-stunted children for the non-verbal reasoning (7.0 ± 3.2vs7.9 ± 3.1; *p* = 0.01) and the school readiness tests (4.3 ± 2.2 vs 3.3 ± 2.1; *p* < 0.001). In addition, HAZ was weakly but significantly correlated with the verbal reasoning test (r = 0.13; *p* < 0.01), and WAZ was correlated with the school readiness tests (r = 0.18; *p* < 0.001). However, mean score of underweight and normal weight children or children with wasting and their normal counterparts was not significantly different (*p* > 0.05). Comparison of cognitive scores of the study children by their nutritional status is shown in Fig. 1

**Table 2:** Cognitive scores by children from the Amhara region, Ethiopia, October 2011-May 2012

Variables	n	Minimum	Maximum	Mean (SD)
Similarity	533	1.0	12.0	9.4 (1.8)
Matrix reasoning	541	1.0	18.0	7.5 (3.3)
School readiness	541	0.0	13.6	3.8 (2.8)

SD, Standard Deviation

**Fig. 2:**
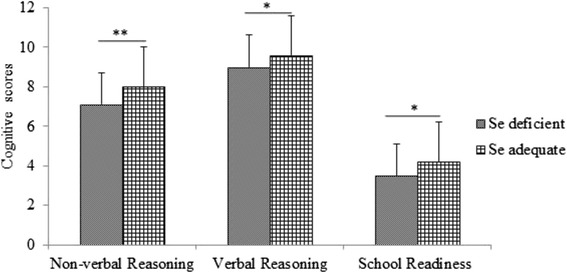
Comparison of cognitive scores between selenium deficient and selenium adequate preschool children.* the difference is significant at *p* < 0.05, **the difference is significant at *p* < 0.01

Preliminary correlations showed that mother’s education, family assets, dietary diversity, iodine status (goiter, urinary iodine, serum thyroxin, serum triodothyronine, and serum thyroglobulin), and serum zinc were not associated with test scores. No sex-related difference with respect to Se status, anthropometric charactertics, iron status, hemoglobin level, or cognitive performance was detected. Compared to non-anemic children, anemic children had significantly lower scores for the similarity test (9.5 ± 1.7 vs 8.9 ± 2.2; *p* = 0.02). However, none of the iron biomarkers had significant associations with cognitive score of the study children (*P* > 0.05). Children with low serum Se had significantly lower cognitive scores for all cognitive tests. Means were 7.1 ± 3.1 vs 8.0 ± 3.2; *p* = 0.005 for the matrix reasoning test; 9.0 ± 1.9 vs 9.6 ± 1.6; *p* = 0.02 for the similarity test; and 3.5 ± 2.4 vs 4.2 ± 2.5; *p* = 0.02 for the school readiness test (Fig. 2). There was no significant difference (*p* > 0.05) between the level of serum Se in stunted and non-stunted or anemic and non-anemic children. Compared to children with one or more micronutrient deficiency (anemia, iron deficiency, Se deficiency) or stunting, normal children had significantly (*p* < 0.05) higher cognitive scores in all of the cognitive tests. In addition, children with coexisting burden of anemia, iron and Se deficiency, and stunting had lower cognitive scores in the similarity and matrix reasoning tests (*p* < 0.05). Children with two or more coexisting nutrient deficiencies scored significantly lower than children affected by single nutrient deficiency in all of the cognitive tests (*p* < 0.05). In addition, hemoglobin in the similarity test (β = 0.32, *p* = 0.001) and serum Se in the matrix reasoning (β = 0.39, *p* = 0.002) and school readiness tests (β = 0.357, *p* = 0.04) were the most important variables to affect cognitive performance of the study children.

## Discussion

This study investigated the association of anthropometric indicators, iron biomarkers, and serum Se with cognitive performance of preschool children from the rural areas of the Amhara region, Ethiopia. The study children had a high prevalence of stunting and Se deficiency. Anemia was a mild public health problem and there was low prevalence of IDA. In the present study children, stunting, Se deficiency, and anemia were associated with poor cognitive performance.

Stunting is the consequence of several macro- and micronutrient deficiencies and poor health. In the present study, stunted children had lower cognitive scores for all of the psychological tests. Similar to the findings of the present study, Mendez & Adair [16] found significantly lower test scores in stunted Filipino children than their non-stunted counterparts. Likewise, in India, stunted children performed poorly and had much lower scores than adequately nourished children on cognitive tests [8]. In addition, in children from developing countries such as Ecuador [28], Vietnam [29], Brazil [30], Malaysia [31], and Cambodia [11], stunting was a significant predictor of lower cognitive test scores. Results of the present study show importantly, that in the year before entering primary school, stunted children are at a disadvantage regarding school readiness and the verbal and nonverbal reasoning skills needed for education in early grades.

According to the Cost of Hunger Study [32], in Ethiopia, 16 % of repetitions in primary school was associated with stunting and stunted children achieve 1.1 years less in school education compared to their normal counterparts. In addition, in that study, it was estimated that child stunting in Ethiopia costs about 16.2 % of the country’s Gross Domestic Product suggesting the enormous challenge of child undernutrition on economic and human capital development on the country and the need for appropriate early childhood interventions to halt the cyclic patterns of poverty.

In the present study children, no significant difference was found between cognitive scores of iron deficient and normal children. In addition, none of the iron biomarkers had significant association with cognitive performance of children. However, results from other researchers show that iron deficiency was associated with poor cognitive performance attributed to alteration on dopamine functioning [33, 34], reduced brain cytochrome *c* concentration, lower fatty acid in the brain thus myelination [35], and disrupting neurochemical profile of the hippocampus [36]. Iron may have had weak effects here because most of the children in the present study were iron sufficient. Compared to non-anemic children, anemic children however had significantly lower cognitive scores for the verbal reasoning test. Similarly, other studies also found significant relationships between Hb concentration and cognitive performance in children [14, 37, 38]. However in addition to poor iron status, anemia typically is caused by several socioeconomic conditions and dietary factors [39] thus the difference in cognitive performance of the present study anemic and non-anemic preschool children may not be attributed to iron status alone.

Selenium levels in serum were found to be associated with all three cognitive measures. This study makes a novel contribution to the literature as there is a dearth of studies on the role of Se in cognitive development of children. The brain is one of the organs with high concentration of Se. In addition, in cases of Se deficiency, it is the organ that remains Se replete the longest. It is also the first organ to obtain adequate level of Se during dietary intake suggesting the important role of Se in brain functions [40]. Several seleno-proteins such as GPx, selenoprotein P, selenoprotein W, and thioredoxin reductase were detected in the brain that modulate and support the activity of the brain by protecting it against oxidative damage [41, 42]. Brain oxidative stress is a casual factor for cognitive impairment [19] and protection against the risk of cognitive deficits by selenoproteins is associated with their role in the antioxidation system [17]. In addition, the effect of Se on cognition is mediated through its role on thyroid metabolism [18].

Although a substantial body of evidence has been compiled on the relationship between Se on cognition of adult humans and animals, little deals with children. In elderly Chinese people, Se deficiency was considered as a risk factor for lower cognitive test outcomes [20]. Similarly, in a 9-year cohort study of community-dwelling French elderly, a decrease in plasma Se was significantly associated with a decline in cognitive performance [19]. Another 4 years follow-up study showed that subjects with low level of Se had an increased risk of cognitive decline [43]. In addition, Se deficiency independently was associated with lower cognitive performance [21]. In rural Bangladesh, maternal red cell Se status during pregnancy was positively associated with children’s developmental measures such as language comprehension and psychomotor development indicating the influence of prenatal Se nutrition on children’s psychomotor development [44].

## Conclusion

In general, the present study children had high prevalence of chronic and acute undernutrition and Se deficiency, mild anemia but low iron deficiency and IDA. Stunting, anemia and Se deficiency were associated with poor cognitive performance. Therefore, identifying the etiological factors and designing appropriate interventions for accelerated stunting reduction and anemia control is vital. The present study children were from subsistence farming households and consume foods grown in their local area. In such settings, Se status of populations is mainly dictated by the level of available Se in the soil [45] which again is governed by the chemistry of the soil [46]. Thus, identifying main soil physicochemical factors that influence Se phytoavailability and knowledge of soil Se concentration would be an important step to design an intervention aimed at increasing Se concentration in staple crops and control Se deficiency. The present study children were from households of similar educational status and occupation. However, there is ample evidence that children’s cognitive outcome can be affected by other factors that were not captured in this analysis, including maternal depression and poverty [47], poor care and inadequate cognitive stimulation [48], parenting style [49], and parasitic infection [50]. In addition, it has been reported that the physical home environment such as housing conditions, risk of exposure to toxicants, chronic and acute noise exposure, and crowding can significantly affect the cognitive performance of children [51].

## Abbreviations

AGP: α_1_-acid glycoprotein
ECLIA: Electrochemilumenescense Immuno Assay
HAZ: height-for-age z-score
IDA: Iron deficiency anemia
sTFR: soluble transferrin receptor
WAZ: weight-for-age z-score
WHO: World Health Organization
WHZ: weight-for-height z-score
WPPSI: Wechsler Preschool and Primary Scale of Intelligence
